# Overcoming disciplinary divides in scientific collaboration: challenges and pathways

**DOI:** 10.3389/fpsyg.2026.1724127

**Published:** 2026-02-04

**Authors:** Jordan A. Johnson, Marlee J. Moore, Madison B. Washam, Allison M. Traylor

**Affiliations:** Department of Psychology, Clemson University, Clemson, SC, United States

**Keywords:** interdisciplinary collaboration, knowledge integration, team dynamics, team process, team science

## Abstract

Interdisciplinary science teams combine diverse expertise to address complex societal challenges and are increasingly vital for advancing research and innovation. However, differences between team members can hinder effective collaboration in these teams. This mini review examines common challenges in interdisciplinary science teams and outlines strategies from team science to address them. We highlight five challenges that undermine team effectiveness: communication across disciplinary boundaries, developing shared mental models and role clarity, navigating hierarchies and status differences, building shared affect and trust, and efficiently using and distributing knowledge. The review includes different recommendations for managing interdisciplinary teamwork, emphasizing evidence-based practices that promote integration, collaboration, and long-term team success.

## Introduction

Interdisciplinary scientific teams (i.e., science teams) are essential for addressing complex societal problems (Wu et al., 2019). Science teams bring together experts from multiple fields to generate new scientific knowledge (Hall et al., 2018; Stokols et al., 2008). Although interdisciplinary collaboration can spark innovation (Andalón et al., 2024), disciplinary diversity introduces organizational, social, and psychological difficulties. Team members differ in research questions, methods, and jargon (Bracken and Oughton, 2006), which can hinder mutual understanding. Differences in epistemological assumptions further complicate cohesion (Joshi and Neely, 2018). Simply assembling experts is insufficient; teams must intentionally manage these differences to collaborate effectively (Salazar et al., 2012).

Emergent team states, such as trust, psychological safety, and shared understanding, may develop unevenly, especially when team members hold different roles or levels of authority (de Jong et al., 2016, 2021; Park et al., 2020). Power and status disparities can shape whose expertise is prioritized (Nembhard and Edmondson, 2006; Xu et al., 2022) and are often reinforced by funding structures, as disciplines that secure larger grants may disproportionately influence project direction and resource allocation (National Research Council, 2015). Teams lacking clear roles or integrative procedures may also struggle to build shared mental models, undermining coordination and productivity (Mathieu et al., 2018).

This mini review identifies core challenges facing science teams and offers evidence-based recommendations to strengthen interdisciplinary collaboration. We synthesize research on interdisciplinary teamwork and team development to highlight five key barriers and outline actionable strategies for improving integrative capacity and collaborative success. Importantly, this review focuses on science teams rather than other types of interdisciplinary teams because scientific work involves discipline-specific publication norms, data standards, and funding structures. As a result, science teams face coordination and communication barriers that are more pronounced than in other interdisciplinary settings (e.g., policy, industry-academic collaborations). Research tasks also require tightly coupled workflows, shared methodological rigor, and integrative theoretical framing, making cross-disciplinary alignment especially challenging. Although our examples draw primarily from scientific research teams, the recommendations in our review offer insights that generalize beyond science teams to other types of interdisciplinary teams.

## Review approach and scope

This mini review adopts a purposeful, narrative synthesis approach to identify core challenges and practical strategies relevant to interdisciplinary science teams. Rather than conducting a formal systematic review, we focused on integrating influential conceptual frameworks, empirical findings, and applied guidance from the science of team science literature (Hall et al., 2018; Stokols et al., 2008). To identify focal themes, we reviewed seminal articles on interdisciplinary collaboration, prior integrative reviews, and applied reports from the National Academies of Sciences and the National Research Council that address the organization, coordination, and funding of interdisciplinary scientific research (e.g., Hall et al., 2018; Salazar et al., 2012; Bammer et al., 2020).

Literature was identified through iterative searches of major databases (e.g., PsycINFO, Web of Science, Google Scholar) using keywords such as interdisciplinary teams, science teams, team cognition, knowledge integration, and collaboration in science, supplemented by backward citation tracking. The five focal challenges were derived through an iterative synthesis process in which recurring coordination, cognitive, and social challenges were compared across sources and refined through discussion among the authors. The resulting challenges reflect issues that consistently emerge across diverse scientific domains and team structures and have direct implications for practice, consistent with prior narrative reviews in team science (Hall et al., 2018; Salazar et al., 2012).

Where prior reviews of interdisciplinary science teams have primarily synthesized evidence across broad analytic domains such as team composition, processes, and institutional influences (e.g., Hall et al., 2018; Stokols et al., 2008), the present review is intentionally structured as a challenge-driven, practice-oriented guide that links common collaboration breakdowns in science teams to concrete tools and leadership practices teams can use to improve integration.

## A primer on collaboration in science teams

Although there is robust evidence that scientific collaborations that span disciplinary, institutional, and geographic boundaries have greater productivity and scientific impact, these differences make it especially difficult for teams to integrate knowledge and develop shared affect and cognition (Hall et al., 2018). Team composition, or the configuration of member attributes, fundamentally shapes how teams work together (Bell et al., 2018). Broadly, research on science teams suggests that when a team’s composition is diverse, the potential for productivity and innovation is higher; however, these teams may also encounter challenges related to integrating ideas across differences (Hall et al., 2018).

Effective knowledge integration in science teams, which relies on team members’ ability to develop shared attitudes, behaviors, and cognition, is necessary to harness the potential of interdisciplinary teams (Bammer et al., 2020; Mohammed et al., 2023). Differences between team members can make developing shared attitudes, behaviors, and cognition difficult. For example, when social scientists and engineers collaborate, disciplinary differences in norms for team interaction may lead team members to develop different perceptions of group processes. When team members do not share perceptions of group processes, they may struggle to accurately predict group interactions and cooperate more effectively (Cannon-Bowers and Salas, 1990). Indeed, when team members disagree on perceptions of group processes, interpersonal interactions are less effective and team performance declines (Mathieu et al., 2000; Mohammed et al., 2010). For example, when some team members perceive group conflict and others do not, it is difficult, if not impossible, for the team to engage in conflict management, yielding lower team performance (Jehn et al., 2010).

Science teams’ integrative capacity can help overcome these challenges. Integrative capacity describes the social and cognitive processes and emergent states that shape a team’s ability to combine diverse knowledge (Salazar et al., 2012). The following sections detail challenges facing science teams, many of which stem from differences in team composition and a team’s ability to form shared attitudes, behaviors, and cognition. We follow this overview by recommending strategies to help teams build their integrative capacity to integrate unique perspectives and increase their innovative potential.

## Challenges of interdisciplinary teams

While collaboration is essential for advancing science, interdisciplinary teams face several barriers that complicate collective work. Scientists from different fields communicate using distinct languages and frameworks, making idea exchange difficult (Star, 2010; Morss et al., 2021; O’Rourke and Crowley, 2013). Teams also struggle to build shared mental models, clarify roles, and align goals (Brower et al., 2021; Mathieu et al., 2018; Paletz and Schunn, 2010; Salazar et al., 2012).

Hierarchical or disciplinary status differences further suppress diverse viewpoints and hinder innovation (Nembhard and Edmondson, 2006). These hierarchies are shaped not only by perceived rigor or prestige, but also by field-specific norms for what counts as meaningful output. For example, expectations for research output (e.g., conference abstracts, high-impact publications, or significant data products) vary widely across disciplines. These evaluative differences often coexist with funding inequities, wherein fields such as medicine, engineering, or genomics routinely attract larger grants and stronger infrastructure. As a result, disciplines that control more substantial funding may gain disproportionate influence over project direction, resource allocation, and timelines, reinforcing status hierarchies before collaboration even begins (Tian et al., 2024; National Research Council, 2015).

Teams may also struggle to develop shared affect or cognition, weakening cohesion and trust (Nembhard and Edmondson, 2006; de Jong et al., 2021). Finally, ineffective use and distribution of knowledge can slow progress (Lewis, 2003; Peltokorpi, 2008). The following sections outline five major challenges and recommendations for strengthening collaboration in science teams (see Table 1).

**TABLE 1:** Challenges and recommendations for science teams.

Challenge	Related teamwork constructs	Recommendations	Relevant citations
Challenge 1: Experts communicate differently about their ideas.	Shared understanding; epistemological fluency	• Use boundary objects and structured dialogue to facilitate mutual comprehension • Develop shared glossaries of terms during a chartering process	Star, 2010; Morss et al., 2021; O’Rourke and Crowley, 2013
Challenge 2: Science teams might struggle to achieve shared mental models or role clarity.	Role clarity; task coordination; shared mental models; team cognition	• Foster structured coordination through planning tools • Use team charters to clarify roles and coordinate tasks • Use concept maps; set integrated project timelines	Brower et al., 2021; Mathieu et al., 2018; Paletz and Schunn, 2010; Salazar et al., 2012
Challenge 3: Hierarchies and status differences between disciplines can silence divergent perspectives.	Equity; inclusion; engagement	• Create role-rotation systems and explicitly recognize contributions • Rotate meeting facilitators • Acknowledge expertise in team documents	Nembhard and Edmondson, 2006
Challenge 4: Team members might struggle to develop shared affect or cognition.	Trust; psychological safety	• Implement inclusive leadership and establish shared norms • Model respectful engagement • Actively solicit diverse perspectives	Nembhard and Edmondson, 2006; de Jong et al., 2021
Challenge 5: Teams use and distribute knowledge inefficiently.	Transactive memory systems	• Support peer education and transparent knowledge mapping • Develop “who knows what” matrices • Provide cross-training sessions to team members	Lewis, 2003; Peltokorpi, 2008

**Challenge 1: Experts communicate differently about their ideas.**

Science teams often face challenges related to differences in communication norms and theoretical frameworks. Across scientific disciplines, there are differences in what constitutes valid knowledge, which methodologies are preferred, and which questions are worthwhile (Brister, 2016; Diaz et al., 2023; O’Rourke and Crowley, 2013). For example, sufficient evidence in one field may not be adequate in another. These discrepancies can cause early misunderstandings and misaligned goals. In some cases, these discrepancies may lead to “disciplinary capture,” which occurs when the presumptions of one discipline predominate at the expense of others (Brister, 2016).

Science teams may also face linguistic mismatches: disciplinary jargon and presumptions about common knowledge frequently obscure meaning and make coordination more difficult (Monteiro and Keating, 2009). Research has indicated that in the absence of a deliberate attempt to create a shared vocabulary, conceptual alignment, or mutual understanding of different approaches, teams risk disintegrating or reverting to the prevailing framework (Morss et al., 2021).

Ultimately, interdisciplinary collaboration necessitates dedicated team processes that support integration across knowledge systems, such as appreciating different perspectives, building interpersonal competencies, and creating institutional mechanisms for collaboration (Bammer et al., 2020). Teams can better co-create integrated frameworks that showcase the strengths of all viewpoints when they actively engage with their differences.

**Recommendation 1:** *Develop shared ways of talking and working together.*

To address communication divides, interdisciplinary teams should implement structured dialogue processes, such as facilitated workshops or guided reflection sessions, that surface and reconcile hidden assumptions across disciplines (O’Rourke and Crowley, 2013). For example, a team of engineers and public health scientists might facilitate a discussion to clarify how each discipline defines “risk,” ensuring they work from a shared understanding. Teams should co-develop boundary objects in parallel, including shared conceptual maps, integrated glossaries, or adaptable models, which provide standard anchors while remaining interpretable across disciplinary languages (Star, 2010).

For instance, a climate science team might co-create a shared conceptual map that visually links meteorological constructs, such as “precipitation anomalies” and “atmospheric circulation patterns,” with social science survey measures like “household risk perception” or “community preparedness behaviors.” This kind of map makes explicit how variables from each discipline relate to one another and provides a common reference point for joint analyses. Similarly, in a collaboration between engineers and public health scientists, a structured dialogue session might focus on unpacking how each field defines “risk,” revealing differences between probabilistic modeling and community vulnerability assessments. Combined, these practices foster epistemic fluency, or the ability to navigate and integrate diverse ways of knowing (Morss et al., 2021). Recent research underscores that dialogue and boundary objects function synergistically, enabling teams to transform epistemic conflict into opportunities for integration and creativity (Arthars et al., 2024). Such practices thus represent a scalable pathway for building durable cross-disciplinary understanding.

**Challenge 2: Unclear roles and expectations confuse teams.**

Interdisciplinary science teams may often struggle to develop shared mental models, or collective understandings of goals, processes, and responsibilities necessary for coordinated action (Mathieu et al., 2000). Shared mental models are critical for taskwork and teamwork (Mathieu et al., 2000); however, their development is complicated by disciplinary differences in terminology, workflow expectations, and problem framing. To build alignment, teams must invest early in structured coordination to support mutual understanding and innovation across disciplinary boundaries (Paletz and Schunn, 2010). For example, a biostatistician in a biomedical research team may expect months of data collection before analysis, while a clinician anticipates quick preliminary results to guide patient trials. These differing assumptions can create frustration and delay progress without a shared mental model. Unlike teams representing a single discipline, whose members typically align in how they approach their work due to similar training, interdisciplinary science teams begin with divergent assumptions and different understandings of how a task should be accomplished. Without early alignment, these differences can lead to miscommunication, redundant or neglected work, and conflict over priorities (Tjosvold, 2008).

**Recommendation 2:** *Set clear roles and shared understanding from the start.*

Science teams can implement structured planning tools to foster team cognition and role clarity, essential for effective coordination (Gorman, 2014). A team charter is a formal document created early in a team’s life cycle that outlines the team’s purpose, roles, goals, and processes (Brower et al., 2021). Team charters support the development of shared mental models by requiring members to communicate expectations for communication, participation, accountability, and conflict management. For example, a neuroscience team might specify in its charter how psychologists and engineers will divide responsibilities for data preprocessing and analysis, reducing duplicate effort.

Alongside charters, concept maps can help teams establish a shared understanding of each other’s perspectives. A concept map is a visual diagram that links key ideas and shows how they relate (Novak and Cañas, 2008). For instance, an environmental health collaboration might develop a concept map connecting “exposure pathways” (toxicology) with “built environment factors” (urban planning) to clarify how each discipline frames the problem. By making disciplinary perspectives visible and negotiable, concept maps strengthen team cognition and support role clarity, especially in early stages of collaboration (Paletz and Schunn, 2010).

**Challenge 3: Power differences may discourage full participation.**

Another persistent challenge in interdisciplinary science teams is the presence of disciplinary hierarchies and status differences. Not all scientific fields, or their representatives, are regarded equally (Cole, 1983). For example, physicians may be granted more authority than public health practitioners, or engineers may dominate discussions with social scientists. These hierarchies emerge from multiple sources, including academic rank, institutional affiliation, funding structures, and broader societal perceptions of rigor (Cole, 1983; Xu et al., 2022).

Importantly, disciplinary hierarchies are often amplified during the proposal and budget development stages of a project. Fields that traditionally receive more substantial external funding, such as clinical sciences, computational disciplines, or engineering, may assume control of budget allocation and resource decisions, positioning their priorities at the center of the collaboration. These funding asymmetries can create structural inequalities before a project even begins, shaping whose work is resourced, whose labor is visible, and whose expertise is viewed as essential (National Research Council, 2015). These power differences are further reinforced by professional identity and demographic histories, with male-dominated or Eurocentric disciplines often afforded greater prestige (Ray, 2019).

Although hierarchy can sometimes streamline decision-making by clarifying authority, unmanaged hierarchies often create inequities that stifle collaboration. Lower-status members may hesitate to share perspectives, while higher-status members may unintentionally dominate conversations, leading to multidisciplinary rather than integrative collaboration. Such dynamics suppress diverse viewpoints and limit innovation by narrowing the scope of ideas considered.

**Recommendation 3:** *Use inclusive leadership to reduce hierarchy barriers.*

Inclusive leadership practices reduce status differences by ensuring all team members, regardless of disciplinary background, have a voice and can make valuable contributions. Practices such as role-rotation systems and rotating meeting facilitation can amplify diverse perspectives through distributing leadership among team members, which equalizes the influence held between disciplines (de Jong et al., 2021). For example, a sustainability research team might rotate meeting facilitation between engineers, ecologists, and social scientists to ensure no single discipline sets the agenda. Explicit recognition can also foster mutual respect and reduce disciplinary status differences by demonstrating how both “high” and “low status” perspectives contribute to the shared team task (Nembhard and Edmondson, 2006; Tannenbaum et al., 2023). For instance, a materials science team may intentionally highlight how qualitative insights from design researchers shape prototype usability, reinforcing that each discipline contributes essential expertise. Finally, acknowledging team members’ unique expertise in team documents (e.g., team charters, RACI matrices) validates their contributions and provides information about knowledge distribution in the team. In this way, role documentation amplifies diverse perspectives by ensuring team members know who to rely on for information outside their expertise (Santos et al., 2021).

**Challenge 4: Teams may struggle to build trust and shared understanding.**

Disciplinary differences and hierarchical structures can also undermine science teams’ ability to build the shared affect and cognition necessary for high performance. Trust often develops inequitably in cross-disciplinary teams, as members are more likely to trust those with similar backgrounds (Costa et al., 2018). As a result, members from dominant disciplines may bond quickly, while those from underrepresented fields risk feeling peripheral. Research on trust asymmetry shows that uneven trust across members reduces cohesion, inhibits information sharing, and harms performance (de Jong et al., 2021; Breuer et al., 2016).

Other affective states, such as psychological safety and conflict, may also emerge unevenly. Psychological safety, the shared belief that it is safe to speak up without fear of ridicule or punishment (Edmondson, 1999), may be distributed unevenly in interdisciplinary teams, particularly disadvantaging those from lower-status disciplines or early in their careers (Nembhard and Edmondson, 2006). Without a shared safety climate, individuals may withhold input outside their domain, diminishing opportunities for integrative collaboration. Conflict patterns are similarly prone to asymmetry, with hidden rifts fragmenting teams when only some members experience tension (Park et al., 2020). Addressing these asymmetries requires intentional practices that create climates of inclusion and safety, which are outlined in the following recommendations.

**Recommendation 4:** *Build trust so everyone feels safe to share ideas.*

Inclusive leadership practices not only reduce status differences between disciplines, but also foster psychological safety and trust, which are essential for open communication, knowledge sharing, and integrative teamwork (Nembhard and Edmondson, 2006). Inclusive leadership practices, such as inviting input from team members, sharing decision-making and leadership functions, and modeling respect engagement across disciplines, can help level disciplinary hierarchies and create psychological safety (Nembhard and Edmondson, 2006). For example, a computational biology team leader might explicitly pause during meetings to ask junior wet-lab researchers whether proposed models align with observed lab realities, signaling that their perspectives matter. Additionally, deliberate practices such as establishing clear expectations for communication, building shared norms, and fostering open dialogue across disciplines contribute meaningfully to trust development in scientific teams (Bennett and Gadlin, 2012). For instance, a geoscience and sociology team might begin meetings with a brief check-in round where each member identifies concerns or uncertainties about the project, normalizing open expression. Finally, sharing leadership influence via practices such as rotating meeting facilitation can support trust development, particularly within diverse teams (de Jong et al., 2021).

**Challenge 5: Teams may use and distribute knowledge inefficiently.**

Interdisciplinary science teams often struggle to use and distribute knowledge efficiently. A central issue is developing accurate awareness of “*who knows what*,” or a transactive memory system. When this system is weak, expertise may be duplicated, overlooked, or misapplied, leading to inefficiencies and missed opportunities for integration (Lewis, 2003; Peltokorpi, 2008). These challenges are magnified in interdisciplinary work, where members rely on different methods, terminologies, and standards of evidence, making it harder to recognize and apply each other’s expertise. As a result, teams may default to familiar collaborators and inadvertently exclude unique perspectives.

Knowledge inefficiencies are especially pronounced in fluid or temporary teams, which have limited time to establish clear patterns of expertise recognition (Ren and Argote, 2011). Without repeated interaction, members may rely on stereotypes of disciplinary skills, creating bottlenecks or redundancies. For example, in a short-term disaster-response team, social scientists may be assumed to handle only surveys while engineers manage modeling, overlooking overlapping competencies that could accelerate integration. Even when knowledge is shared, research shows that sharing alone is insufficient unless the team also develops processes for applying it (Choi et al., 2010). In biomedical collaborations, for instance, data often circulate across labs but go unused without agreed-upon procedures for incorporating them into clinical trials. These challenges highlight the need for intentional practices to help teams map, share, and apply expertise effectively, motivating the following recommendation.

**Recommendation 5:** *Use simple tools to track and share expertise.*

To collaborate effectively, science teams must develop strong transactive memory systems, shared awareness of “who knows what” (Borgatti and Foster, 2003). Peer education about disciplinary roles supports this awareness and prevents coordination breakdowns by helping members locate and access relevant expertise (Lewis, 2003; Peltokorpi, 2008). Tools such as transparent knowledge mapping and collaborative concept mapping clarify how expertise is distributed within the team (Santos et al., 2021). For example, an Arctic climate hazards team might maintain an “expertise matrix” listing members’ methods, such as remote sensing, hydrological modeling, or ethnographic interviewing, to ensure others know whom to consult. These practices help teams organize information, clarify roles, and better understand disciplinary perspectives.

Cross-training offers a second path for strengthening transactive memory systems by exposing members to the tasks and constraints of other roles (Shuffler et al., 2015). For example, in an infectious-disease modeling collaboration, public health researchers may explain how case-report data are validated while modelers demonstrate how those data feed into simulation parameters. Together, knowledge mapping and cross-training promote efficient knowledge distribution by ensuring team members know who holds critical expertise.

## Future directions

Although this mini review synthesizes several recurring challenges in interdisciplinary science teams, important questions remain about how these dynamics evolve in modern, digitally mediated collaboration. Building on distributed cognition and knowledge distribution, future work should clarify when these challenges are most likely to emerge, which coordination mechanisms are most effective for different team structures, and how new tools (e.g., shared digital workspaces and AI-supported workflows) reshape integration, participation, and learning. Table 2 outlines targeted directions for future research that build on this review’s practical, tool-oriented focus and articulate testable design principles for contemporary science teams.

**TABLE 2:** Future research directions for interdisciplinary science teams.

Future research focus	Open question/gap	Example study approach	Practical payoff
Distributed cognition as a unifying mechanism	Do the five challenges reflect a common underlying problem: failures in how teams represent, store, retrieve, and coordinate knowledge across people and tools?	Longitudinal field studies linking artifacts (docs, code repos, dashboards) + interaction traces to integration outcomes	Moves the field toward design principles (what structures/tools to implement, when) rather than general advice
Knowledge distribution and inequity	When does expertise become invisible (or undervalued) due to status, funding, or role structure, and how does that distort decision-making?	Social network + contribution analyses (authorship, meeting talk time, task allocation) paired with surveys of influence/voice	Identifies early warning indicators of exclusion and provides levers to correct course (role rotation, documentation norms, decision rules)
Digitally mediated collaboration	Which collaboration practices work best in remote/hybrid science teams where informal sensemaking is reduced?	Comparative studies of co-located vs. distributed teams; experience sampling of coordination breakdowns	Specifies which practices should be synchronous vs. asynchronous and how to maintain shared mental models at distance
AI-augmented teamwork	When do AI tools (summarizers, coding assistants, literature mappers) improve integration vs. reinforce disciplinary capture or bias?	Experiments varying AI support + domain dominance; auditing AI outputs for framing bias	Helps teams adopt AI in ways that increase epistemic diversity rather than amplifying the loudest discipline
Coordination mechanisms across project phases	Which mechanisms matter most at startup (proposal/design), midstream (execution), and late stage (writing/translation)?	Phase-based designs; repeated measures across milestones; “critical incident” sampling	Produces phase-specific guidance (what to do before problems compound)
Interventions and toolkits	Which lightweight interventions (charters, mapping, facilitation scripts, expertise matrices) reliably improve integration and for whom?	Pragmatic trials in real teams; implementation research (feasibility, adherence, adaptation)	Turns recommendations into validated toolkits that teams can adopt with confidence

## Conclusion

Interdisciplinary teams play a critical role in addressing complex societal problems, but commonly face barriers related to communication, role clarity, hierarchy, and uneven trust (O’Rourke and Crowley, 2013; Nembhard and Edmondson, 2006; de Jong et al., 2021). This mini-review does not aim to catalog all challenges such teams encounter; rather, it synthesizes several core, recurring barriers that hinder integration and collaboration. By outlining evidence-based strategies to address these issues, we highlight practical pathways that can strengthen teamwork, support psychological safety and equity, and ultimately enhance scientific innovation (Bammer et al., 2020; Rolland et al., 2021). To support a more generative research agenda, Table 2 highlights future directions centered on distributed cognition and digitally mediated collaboration that can translate these recurring challenges into testable mechanisms and actionable design principles for science teams.
