# Correction: Overcoming disciplinary divides in scientific collaboration: challenges and pathways

**DOI:** 10.3389/fpsyg.2026.1827593

**Published:** 2026-03-24

**Authors:** Jordan A. Johnson, Marlee J. Moore, Madison B. Washam, Allison M. Traylor

**Affiliations:** Department of Psychology, Clemson University, Clemson, SC, United States

**Keywords:** interdisciplinary collaboration, knowledge integration, team dynamics, team process, team science

In the published article, the funder Clemson University Libraries Open Access Publishing Fund was erroneously omitted.

The correct **Funding** statement reads:

The author(s) declared that financial support was received for this work and/or its publication. The support was provided by the Clemson University Libraries Open Access Publishing Fund.

The original version of this article has been updated.

